# Comparative Study of Expression of Smad3 in Oral Lichen Planus and Normal Oral Mucosa

**Published:** 2013

**Authors:** Shima Nafarzadeh, Samad Ejtehadi, Pouyan Amini Shakib, Majid Fereidooni, Ali Bijani

**Affiliations:** 1 *Department of Oral and Maxillofacial Pathology, Faculty of Dentistry, Babol University of Medical Sciences, Babol, Iran.*; 2 *Private Dentist, Babol, Iran.*; 3 *Department of Periodontology, Faculty of Dentistry, Babol University of Medical Sciences, Babol, Iran.*; 4 *Non-Communicable Pediatric Diseases Research Center, Amirkola Hospital, Babol University of Medical Sciences, Babol, Iran.*

**Keywords:** Oral lichen planus, smad 3, transforming growth factor β

## Abstract

Oral lichen planus (OLP) is a chronic inflammatory disease of the oral mucosa which is considered by the World Health Organization (WHO) as a premalignant condition. One step in malignant development is so called epithelial mesenchymal transition (EMT), a process whereby epithelial cells acquire mesenchymal characteristics. A factor known to induce EMT is the transforming growth factor-β (TGF-β), which uses the Smad proteins as mediators for its signaling. The aim of this study was to compare the expression of Smad 3 in Oral Lichen Planus and normal oral mucosa. This descriptive analytic study was performed on 30 patients with OLP (21 women and 9 men with mean age of 45.23± 2.44 years) and 20 normal oral mucosa (14 women and 6 men with mean age of 46.95± 2.21 years). The samples were studied by immunohistochemical staining. Data were analyzed with paired T-test and Wilcoxon test by SPSS software. Expression of Smad3 in OLP samples and normal oral mucosa was different. This difference was statistically significant (P<0.001). The apparently higher expression of Smad 3 in oral lichen planus compared to normal oral mucosa might help to discuss its higher potential for malignant transition.

Lichen planus (LP) is a relatively common chronic dermatologic disease that often affects the oral mucosa with a prevalence ranging from 0.2 to 4% (1-2). Indeed Oral lichen planus (OLP) is considered as a chronic disease with dynamic evolution (2) for which several panels of diagnostic criteria, such as the modified WHO one, have been proposed to render a more reliable and accurate diagnosis (3). The cause of LP is unknown; it is generally considered to be an immunologically mediated process (4). Hence, there is a questionable theory about the potential of OLP for malignant transformation into oral squamous cell carcinoma (OSCC) (5). Although Gonzalez-Moles et al. (6) have reported the frequency of 0 to 12.5% for this kind of transformation, in a recent study, Shen et al. (7) demonstrated that the incidence of OSCC developing in lesions previously diagnosed as OLP, is less than 1% and they didn’t entirely rule out these cases as de novo OSCCs.

Transforming growth factor β (TGFβ) regulates several cellular processes including proliferation, differentiation, migration and death (8). Numerous studies have demonstrated the importance of TGFβ signaling in cancer progression and metastasis. Although some studies support the tumor suppressive effects of TGFβ, other studies indicate a tumor promoting function for TGFβ. Thus, it seems that this cytokine may have various effects on different stages of tumorogenesis (9).

Smad proteins are an integral part of the TGFβ signaling pathway. After ligand binding, the TGFβ receptor II (TβRII) phosphorylates TGFβ receptor I (TβRI) which consequently phosphorylates and activates two subgroups of Smad proteins (Smad 2 and Smad 3). These Smad proteins bind to a co-Smad and finally the Smad complex translocates to the nucleus, where the transcription of TGFβ-responsive genes are regulated (9).

Some previous studies have demonstrated a significant reduction of Smad 3 in TGFβ signaling pathway in tissues with erythematous OLP compared to normal oral mucosa (10) and some have shown an increased expression of this protein and consequently, related it to early stages of OLP transition to malignancy (11). Therefore, in this study we investigated the immunohistochemical (IHC) expression of Smad3 in tissues with OLP and adjacent normal tissues to determine the relative role of this protein in evolution of OLP and evaluate the prognostic value of this marker when the progression to malignancy is suspected.

## Materials and Methods

**Tissue specimens and clinical data**

Our study consisted of 30 biopsy specimens taken from patients with OLP (24 reticular and 6 erosive), as defined by modified WHO criteria (3) and 20 samples of non-inflammatory, non-precancerous adjacent normal oral epithelium as control group. All cases were retrieved from the files of Oral and Maxillofacial Pathology Department of Dental Faculty of Babol University of Medical Sciences, between 2005 and 2010. All biopsies have been fixed in formalin 10% and processed to paraffin-embedded tissue blocks according to the routine practice. Representative Hematoxylin & Eosin sections were assessed to confirm the diagnosis and sufficing of the tissue for further evaluation. The clinical and demographic data were collected from pre-existing medical records.

**Immunohistochemistry**

Smad3 expression was assessed by immuno-histochemical analysis using streptavidin-biotin-peroxidase technique. Formalin-fixed paraffin embedded tissue samples were cut into 4 µm-thick sections and mounted on silane-coated slides. The sections were deparaffinized with xylene, rehydra-ted in graded ethanol and immersed in 0.3% H2O2 in methanol for 10 min at room temperature (RT) to block the endogenous peroxidase activity. To retrieve the antigen, the slides were treated using microwav in 0.01 mol/L citrate buffer (PH= 6.2) at 95^o^C for 15 min and then washed in distilled water. The sections were then incubated with primary antibody (ab55479, Abcam Corp., Cambridge, UK) for 60 min. For negative control, no primary antibody was added. After washing in PBS, the sections were incubated with Horseradish peroxidase (HRP) conjugated secondary antibody at RT for 30 min. Following PBS washing, sections were incubated with 3, 3' diaminobenzidine tetra-hydrochloride (DAB) (Dako, Corp, Denmark) for 10 min. Finally, the sections were counterstained with hematoxylin and then dehydrated, cleared and covered with a coverslip. Also, colorectal carci-noma specimens were used as positive controls (based on related data sheet) to allow antibody expression comparison.

**Evaluation of Immunohistochemical staining**

The cytoplasmic and / or nuclear immuno-histochemical expression of Smad 3 was evaluated quantitatively using a light microscopy according to what Danielsson et al. performed in their study (11). According to the staining intensity (weak to severe), the percentage of stained epithelial cells in the area involved by OLP and adjacent normal tissue was determined and the tissues were classified into four groups: (0) 0%; (1) 1 to 25%; (2) 26 to 50%; (3) 51 to 75%; (4) higher than 75 %; at a magnification of 100×.

Finally, paired T-test and Wilcoxon test were used to assess the statistical significance of the correlations between Smad3 expression and the health status of the tissue and P<0.05 was considered significant.

## Results

 The mean age of the patients with OLP and control group were 45.23 years (range 23 to 75) and 46.95 years (range 28 to 75), respectively. Twenty one (70%) of the patients with OLP and fourteen (70%) of the control group were women. The remaining thirty percent of both groups were men.

**Table 1:** IHC staining findings of Smad3 in OLP and control group

**Group**	**Case (n)/ (%)**	**Control (n)/ (%)**
0	0/0	3/15
1	3/10	17/85
2	14/46.7	0/0
3	4/13.3	0/0
4	9/30	0/0

**Fig 1:**
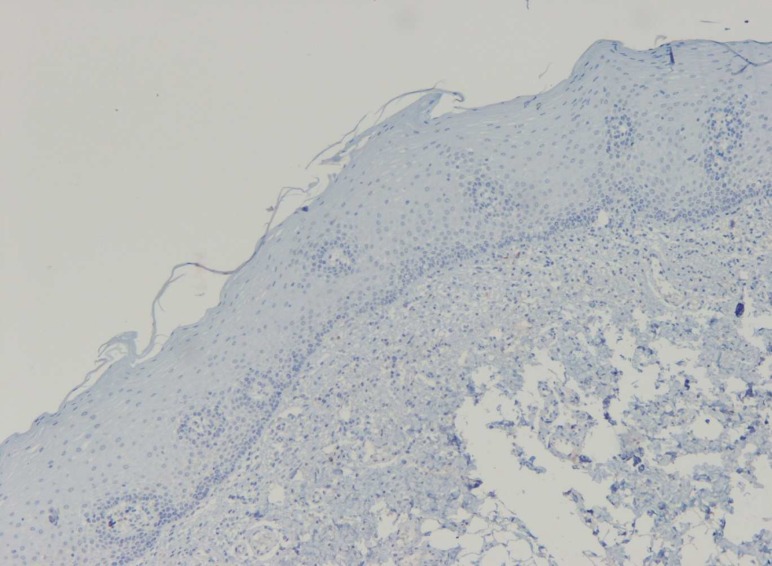
IHC staining of normal oral mucosa showing no expression of SMAD3 (X100).

**Fig 2:**
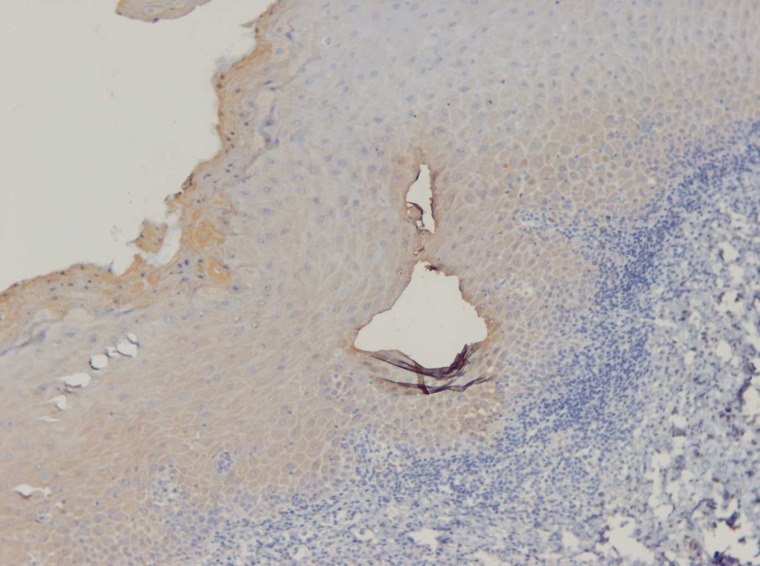
IHC staining of OLP demonstrates high expression (score 4) of SMAD3 (X400).

22 (73.4%) samples of lichen planus were located in buccal mucosa, 1 (3.3%) in labial mucosa, 4 (13.3%) in tongue, 2 (6.7) in gingiva and 1 (3.3) in palate. In normal oral mucosa, the distribution of samples in the above locations was 16 (80%), 1 (5%), 2 (10%), 1 (5%) and 0 (0%) respectively. The results of immunohistochemical staining of Smad3 in both case and control groups are represented in table 1 and figures 1 and 2. Also, a significant difference of the expression of Smad3 was observed between normal oral mucosa and OLP (Table 2). 

## Discussion

We designed the present study in order to evaluate Epithelial-Mesenchymal Transition (EMT) changes in oral lichen planus because of malignant behavior of this disease and its tendency to change to head and neck squamous cell carcinoma (HNSCC) which was found in previous studies.

 Among the proteins which have role in EMT, we chose Smad3 because it is involved in TGF-β pathway and is highly expressed in several cancers (12). The SMAD genes encode components of the TGF-β signaling normally inhibits the cell cycle; the loss of these genes may allow unrestrained cell growth (4). Smad3 together with co-factor SNAIL also acts in repression of E-cadherin and Occludin which help tumoral progression (13). Smad3 may have a role in apoptosis and also in inflammation, but the results are contradictory (14-15). 

**Table 2:** Comparison of immunohistochemichal expression of Smad3 in OLP and normal oral mucosa

**Sample type**	**Mean value**	**Std. deviation**	**T- test**	**Wilcoxon**
*OLP*	64.5	30.17	P<0.001	P=0.001
*Normal oral mucosa*	11.25	6.85		

In this study, we found a higher expression rate in OLP compared to normal oral mucosa which was statistically significant (P<0.001). Different target genes are regulated by Smad 2 and Smad 3 (16). So, the higher expression of Smad 3 in OLP could prove that Smad 3 target genes are more expressed in OLP. This finding supports the malignant potential of OLP.

Karatsaidis et al. found a decreased expression of Smad 2/3 in their study. This difference could be due to the use of different antibodies, different IHC methods and inclusion of both OLP and oral lichenoid reactions in their research (10).

Danielsson et al. found an increased expression of Smad 3 in OLP, dysplasia and tumors compared to normal controls which is in accordance with our findings (11).

In the present study, expression was seen in the nucleus of epithelial cells which is explained by Smad3 active status due to phosphorylation. Considering normal oral mucosa, we found few cells with Smad 3 expression which is in accor-dance with Danielson et al.'s study results (11), but is in contrast to the study of Karatsaidis who found a strong expression of Smad 3 in normal oral mucosa (10).

As a whole, our findings showed an increased expression of Smad 3 in OLP compared to normal oral mucosa which might be due to its probable role in apoptosis, inflammation and EMT and may indicate its higher potential for malignant transition.
